# Reply to the Criticisms of a Patient

**Published:** 1914-08-08

**Authors:** 


### THE EDITOR'S LETTER-BOX.

### "Reply to the Criticisms of a Patient."

*To the Editor of* The Hospital.

Sir,—In your present issue I notice an article which you describe "as a protest against the 'Patients' Point of View,'" which appeared in The Hospital of June 20. It should be unnecessary to state that I am glad to have my description examined by nurses, but it is disappointing to find that not one of my points has been specifically dealt with. I know, of course, that the article by the nurses expands itself into a description of the thoughtlessness of the average convalescent patient, but as it begins by professing to "offer some defence of what the paying patient considers 'faults of administration,'" it ought to deal with these faults themselves. It does not once touch them.

The main "fault of administration" alleged by me was, and is, that in the early morning, when everyone was wanting them, there were not enough bed-pans to go round. That is a perfectly simple direct complaint. It was followed by the question, "Why could not one be assigned to each room?" Will the writer of the protest please explain why not? A second point was that "at this, *the regularly busiest time of every day*," one could not get attended to because the nurses were too busy—that is, too few in number—to answer the bells. I can quite imagine, with the protesting nurses, that "this means of communication is shamefully abused"; but that does not alter the fact that if bells are installed at all, arrangements, especially at the busiest hour of every day, must be made for answering them. What has the writer to object to in this? The rest of the protest is really "tit-for-tat" in the form of an argument; and no doubt most of the faults that can be urged against nurses can be urged equally against patients; but these general indictments of sick human nature are no answer to specific criticisms. What does carry us far—disastrously far, it seems to me—is the following comment, which is really the keynote of the whole reply: "The patient," write the protesting nurses, "who pays two or three guineas a week for treatment, surgical operations, food, room, nursing, and medical attendance, inclusive, is undoubtedly receiving charity, and has no right to expect the same trained attention as he might demand at an expensive nursing home." What an excuse!

This is the attitude of the workhouse master to the inmates of a Poor-Law institution. That it should be put forward by hospital nurses as the spirit animating the relation of staff to patient in a pay-ward of a voluntary hospital is extremely surprising. If this is the voluntary spirit of charity, it is indistinguishable from that of the old Poor Law, for its assumption is that to receive charity is disgraceful. That is not the Christian view. As a matter of fact, the pay-ward, like the rest of a voluntary hospital, is appealed for to subscribers on the plea that it gives the best which can be given just *because* of the patient's inability to pay. The result is that those who are acquainted with both know that any part of a voluntary hospital is a better place to be ill in than the average "expensive nursing home," which (as the protesters carefully point out) has not been built, but only "adapted more or less," for nursing sick patients. The hospital standard, both of spirit and administration, is naturally often lacking in these professedly commercial institutions; but their staffs have not the right—and as my recent experience proved, not the justification—to imagine that power to pay is the ruling factor in a voluntary hospital pay-ward. Having been reminded of this, the matter must end, so far as I am concerned, with my once again repeating how sincerely grateful I am for all the kindness and skilled attention I received. I should feel fortunate to be there again were I ill. I had no criticism to make except as to bed-pans and bells.—Yours faithfully, The Patient.

